# A Robust Dynamic Heart-Rate Detection Algorithm Framework During Intense Physical Activities Using Photoplethysmographic Signals

**DOI:** 10.3390/s17112450

**Published:** 2017-10-25

**Authors:** Jiajia Song, Dan Li, Xiaoyuan Ma, Guowei Teng, Jianming Wei

**Affiliations:** 1 School of Communication and Information Engineering, Shanghai University, Shanghai 200444, China; songjiajia@sari.ac.cn (J.S.); tenggw@shu.edu.cn (G.T.); 2 Shanghai Advanced Research Institute, Chinese Academy of Sciences, Shanghai 201210, China; maxy@sari.ac.cn (X.M.); wjm@sari.ac.cn (J.W.); 3 University of Chinese Academy of Sciences, Beijing 100049, China

**Keywords:** photoplethysmography, motion artifacts, heart rate estimation, intense physical activities, the single notch filter, EEMD

## Abstract

Dynamic accurate heart-rate (HR) estimation using a photoplethysmogram (PPG) during intense physical activities is always challenging due to corruption by motion artifacts (MAs). It is difficult to reconstruct a clean signal and extract HR from contaminated PPG. This paper proposes a robust HR-estimation algorithm framework that uses one-channel PPG and tri-axis acceleration data to reconstruct the PPG and calculate the HR based on features of the PPG and spectral analysis. Firstly, the signal is judged by the presence of MAs. Then, the spectral peaks corresponding to acceleration data are filtered from the periodogram of the PPG when MAs exist. Different signal-processing methods are applied based on the amount of remaining PPG spectral peaks. The main MA-removal algorithm (NFEEMD) includes the repeated single-notch filter and ensemble empirical mode decomposition. Finally, HR calibration is designed to ensure the accuracy of HR tracking. The NFEEMD algorithm was performed on the 23 datasets from the 2015 IEEE Signal Processing Cup Database. The average estimation errors were 1.12 BPM (12 training datasets), 2.63 BPM (10 testing datasets) and 1.87 BPM (all 23 datasets), respectively. The Pearson correlation was 0.992. The experiment results illustrate that the proposed algorithm is not only suitable for HR estimation during continuous activities, like slow running (13 training datasets), but also for intense physical activities with acceleration, like arm exercise (10 testing datasets).

## 1. Introduction

Photoplethysmography (PPG) is a kind of popular optical measurement technique that can be used to detect blood volume changes in the micro-vascular bed of tissue [1]. Because of its simplicity and low-cost advantages, PPG has become the new technique for wearable measurement instead of the conventional electrocardiography (ECG) technique. The pulsatile “AC” physiological waveform can be obtained due to cardiac synchronous changes in blood volume with the heartbeat. Due to this property, PPG can be a source of real-time heart rate (HR) information calculation [2]. Ear, finger and wrist are all optional PPG measurement sites. However, in measurement sites, noise interference produced by motion artifacts (MAs) and cardiac arrhythmia is inevitable. Due to human movement, relative motion may occur between the sensor and skin so that the principle component of true HR information is weakened. The quality of the PPG sensor signal is especially susceptible to motion artifacts. In other words, the accuracy of heart rate estimation depends on the quality of the PPG. Therefore, it is essential to minimize the influence of motion artifacts in order to improve the accuracy of heart rate estimation.

Several methods are usually used to recover or reconstruct a clean PPG signal from a corrupted one before HR is extracted. Generally speaking, there are five kinds of these MA-removal algorithms: classical digital filters [3]; adaptive filters [4]; time-frequency analysis (wavelet decomposition [5], singular value decomposition [6], and empirical mode decomposition [7]); spectrum analysis; and bland signal processing [8].

Those algorithms mentioned above can be applied to the signals which are corrupted slightly when motion artifacts are not strong. However, these techniques cannot figure out the precise heart rate estimation when intense physical exercise such as boxing occurs. So, people would always prefer to use complex algorithms when extracting HR from the corrupted PPG signals, rather than a single technique. When the motion artifacts are strong, heart-rate information can be mostly masked by the noise component. Thus, the removal of MAs in intense exercise from the PPG is always challenging. Fukushima et al. [9] and Zhang et al. [10] argue that acceleration data are also helpful for removing MAs. In this paper, tri-axis acceleration data play an important role in MA removal.

Recently, some study groups have concentrated on the solution of strong MA removal and have made progress. Therefore, many state-of-the-art algorithms are proposed. Zhang et al. [11] put forward the TROIKA framework which consists of three key parts, namely signal decomposition, sparse signal reconstruction, and spectral peak tracking. In this framework, signal decomposition using singular spectrum analysis is applied to cancel partial MAs. Sparse PPG signal reconstruction puts the sparse signal into a high-solution spectrum so that the true peak corresponding to the heart rate is found. Then, Zhang et al. proposed an improved algorithm JOSS [10] with the help of acceleration data. The spectra of PPG signals and simultaneous acceleration signals are jointly estimated using the multiple measurement vector (MMV) model in sparse signal recovery. This algorithm shows the effect of acceleration data on the accuracy of heart-rate estimation from the PPG. However, on the one hand, the sparse signal reconstruction entails high computational complexity and will take a long time in practical use. On the other hand, all sliding windows in the two methods mentioned above must experience every step, which can result in a complex algorithm and a high computational cost. In order to save computational cost, for windows that indicate MAs are not too significant an influence upon finding the true peak of the HR with acceleration data, there is no need to go through every step.

The SpaMA algorithm [12] proposed by Salehizadeh et al. combines the PPG signal and acceleration data. Its key idea is to calculate the power spectral density of both PPG and acceleration data, and the related frequency peaks resulting from MAs can be distinguished from the PPG spectrum. This method performs better in the first 12 datasets, but the off-track error is large in other datasets that have stronger MAs during intense arm movements. Another algorithm named CNAFSD [13] proposed a hybrid-motion artifacts-removal method, which combines non-linear adaptive filtering and signal decomposition (singular spectrum analysis). Sun et al. proposed SPECTRAP [14] using a new spectrum-subtraction algorithm. Another algorithm, WFPV [15], uses a Wiener filter to suppress MAs and a phase vocoder to improve the HR estimate. The algorithms mentioned above apply every step for all sliding windows so that the calculation and run time are relatively large. Therefore, it is essential to make multilevel decisions according to the different features of the PPG.

**Figure 1:**
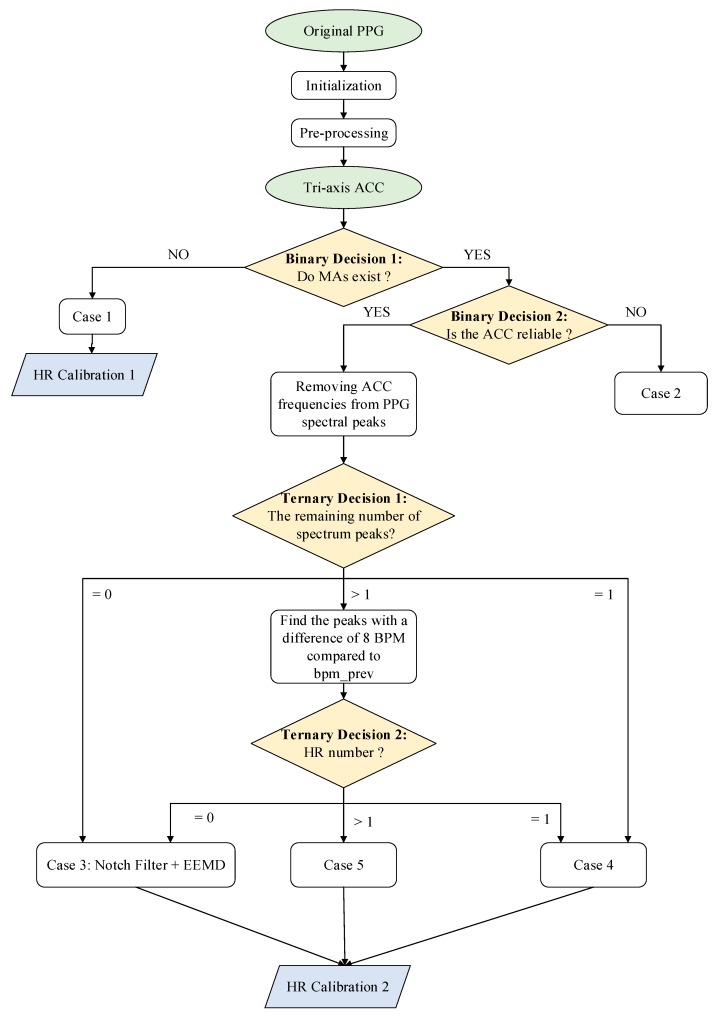
The overall flowchart of the NFEEMD algorithm.

In this paper, a new approach combining the single notch filter and improved EEMD (NFEEMD) is proposed to estimate HR, during intense exercise, from the PPG. Acceleration data are adopted to detect if MAs exist and provide several reliable spectral peaks corresponding to MAs. The proposed algorithm framework determines the different signal-processing methods for different PPG signals by designing multiple decisions. Two binary decisions related to acceleration data and two ternary decisions related to the number of spectral peaks are applied to the NFEEMD algorithm. Our proposed algorithm is different from the previous one, which uses sequential execution for every sliding window. This approach consists of three stages (Figure 1). Stage 1 aims to find out whether the original PPG signal is seriously corrupted by MAs and then chooses one way to estimate the HR by a precise binary-decision algorithm. When there are no significant MAs, HR can be obtained from the pre-processed signal spectrum peaks. When there are significant MAs, then stage 2 appears to work. Stage 2 removes the corresponding MA spectrum peaks from pre-processed signal spectrum peaks and then goes through the next step combined with the single notch filter and the improved EEMD by a ternary-decision algorithm. Every step has one kind of HR tracking method. With the purpose of avoiding spectrum peak loss to keep tracking valid, stage 3 will calibrate the value of HR.

The main contribution of this algorithm has two aspects: (1) The different signal processing methods with high estimation accuracy are proposed according to different PPG features instead of using the sequential execution mode for every sliding window. (2) The running time in real-time HR estimation is greatly reduced, due to reduced computational complexity.

This paper is organized as follows: Section 2 discusses various conditions where there is very strong MAs so that HR estimation is difficult. Section 3 introduces the proposed algorithm framework to deal with the problem mentioned above. Section 4 illustrates the experimental results using 23 datasets and compares these with other framework algorithms in this field, with corresponding discussions. Moreover, all datasets used in this paper will be available at Zhilin Zhang’s homepage: https://sites.google.com/site/researchbyzhang/ and the related paper is [11].

## 2. Materials and Methods

In this section, the complete NFEEMD algorithm framework will be mentioned and the corresponding details of the proposed algorithm will be given. The overall flowchart of NFEEMD could be shown in Figure 1.

The framework of HR estimation during intense physical exercise is presented in Figure 1, then the signal-processing method in every case will be illustrated. For this algorithm, one-channel raw PPG and tri-axis acceleration data are needed. This framework includes two binary decisions and two ternary decisions.

**Initialization:** In the initialization stage, the system can enter into a stable heart-rate tracking state if HR can be estimated by choosing only one spectrum peak from the raw PPG periodogram. Before the stable heart-rate tracking state, the estimated HR values can be discarded, since the contaminated PPG with many spectral peaks may provide wrong prior HR information initially. It is known that the HR value cannot change rapidly in a short time [16], therefore correct prior HR information is essential to maintain continuous HR tracking. Once stable tracking starts, the first HR value is obtained. Then the main algorithm uses the previous HR estimation *bpm_prev* as its base. It should be noted that we identify the spectral peaks if they are larger than 30% of the maximum amplitude in the periodogram.

**Pre-processing:** It is necessary for raw PPG to be pre-processed in order to highlight the HR information. The repeated moving average filter [3] is applied to obtain the signal component of 0.5–3 Hz. The pre-processing signal is used in two cases: (1) in the HR tracking when there are no MAs; (2) in the first ternary decision for finding the number of PPG spectral peaks when there are MAs.

**Figure 2:**
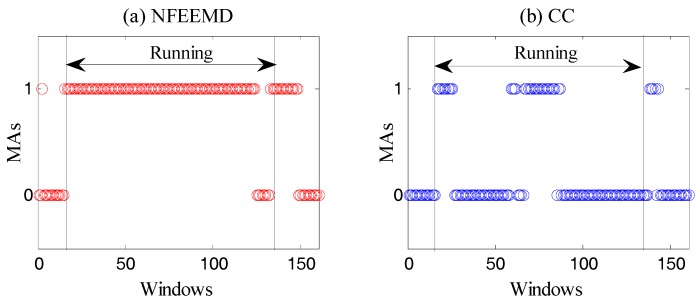
The MAs’ existence judgment algorithm comparison on dataset 8. The subject is in a running state between the 16 and 135 windows, and in a rest state in the remaining windows.

**Binary Decision 1:** This step aims to determine if there are MAs through the amplitude of the acceleration periodogram. MAs exist when the spectral amplitude of every axis acceleration data is greater than 0.1 (power spectral density). Of course, there are other algorithms for determining the existence of MAs, such as the correlation coefficient (CC) [17]. Through the comparison of three methods, the resulting judgement can be described in Figure 2.

It is known that there must be motion artifacts in the running state. Moreover, PPG and MAs may overlap when MAs are very strong. From Figure 2, it is obvious that our method is more suitable for the experiment than the CC algorithm.

**Case 1:** When MAs do not exist, an HR-estimation process is proposed. *bpm_track* (the final estimated HR value) depends on the highest spectral peak of pre-processed PPG. If |*bpm_track* − *bpm_prev*| > *th_pass* (a constant threshold), *bpm_track* is equal to *bpm_prev*.

**HR Calibration 1:** According to the calculation rules above, an HR-calibration mechanism is designed in order to prevent the correct spectral peak being lost due to over-reliance on the past heart rate values. If two consecutive heart-rate values are dependent on the past, the **HR Calibration 1** algorithm will start when MAs do not exist. In this condition, *bpm_track* will be recalculated through the original PPG signal. Meanwhile, if |*bpm_track* − *bpm_prev*| ≤ *th_pass*, the value of *bpm_track* will be replaced by the *bpm_prev’s*.

**Binary Decision 2:** The purpose of this step is to determine whether the accelerometer signal is reliable and whether the frequency component associated with motion artifacts can be accurately provided during strenuous exercise. It is known that the spectrum of tri-axis acceleration data is too messy to accurately extract the MA component by exploring the acceleration data. If the number of spectral peaks in any axis accelerometer data exceeds *th_acc_peaks* (a constant), then we consider the acceleration data of this window to be unreliable.

**Case 2:** At this time, *bpm_track* will be replaced by past HR value.

After **Binary Decision 2**, if the acceleration data is reliable, then we will remove the spectral peaks (absolute value less than or equal to 8) associated with the tri-axis acceleration from the spectral peaks of pre-processed PPG. The number of remaining spectral peak is then counted. **Ternary Decision 1** divides the situation into three categories: the remaining zero (the peak corresponding to HR cannot be detected), the remaining one, leaving more than one. For the second case, a **Ternary Decision 2** was designed by counting the peak number with a difference of *th_pass* BPM compared to *bpm_prev*. This ternary decision also divides the situation into three categories: the remaining zero, the remaining one, leaving more than one. As shown in Figure 1, the same situation undergoes the same treatment.

**Case 3:** (the remaining zero): This is the main part of the algorithm used to remove motion artifacts. The repeated single notch filter and improved EEMD algorithm are adopted herein.

(1) Single notch filter

A second-order IIR filter is used to implement the single notch filter here, whose amplitude response is zero at a certain frequency. It is usually used to eliminate a particular frequency component, such as the removal of 50 Hz power-line interference. In this paper, the single notch filter is applied to the motion artifacts’ removal from the PPG signal spectrum peaks based on the tri-axis acceleration signal spectrum peaks. The system function of the single notch filter is defined in Equation (1) as follows:

(1) $$H(z)=\frac{1-(2\cos w_{0})z^{-1}+z^{-2}}{1-(2\cos w_{0})rz^{-1}+r^{2}z^{-2}}$$

where $w_{0}=2\pi f_{0}/f_{s}$ is the notch digital frequency (rad); $f_{0}$ is notch frequency (Hz); $f_{s}$ is sampling frequency; and *r* is a constant (*r* = 0.96). The single notch filter can be applied when the MAs of the original signal exist after the binary decision. According to the tri-axis acceleration data, several frequencies corresponding to MAs are identified through the periodogram. Then, the related frequencies in the original PPG signal are removed, and the reconstructed, clean signal is obtained.

**Figure 3:**
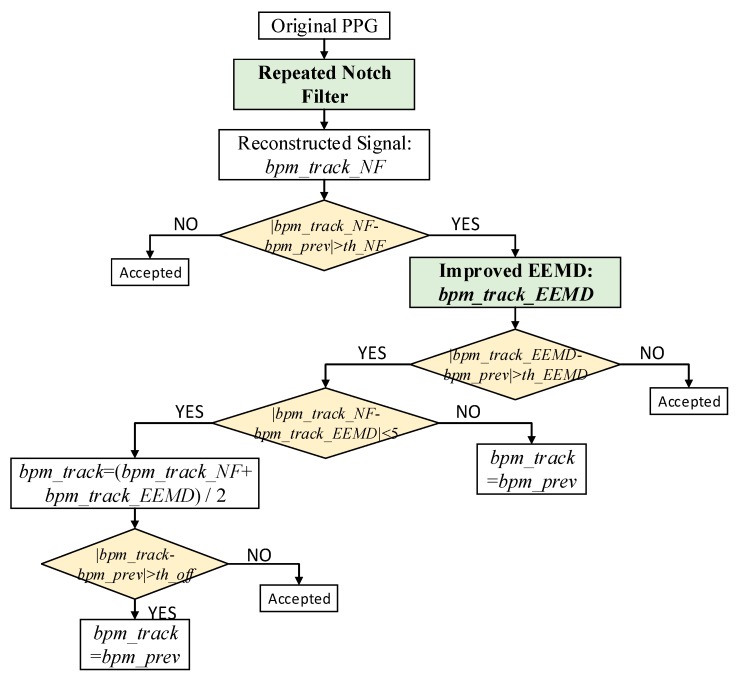
The flowchart of the main part of the NFEEMD algorithm for removing motion artifacts.

Figure 3 shows a flowchart depicting the removal of MAs to reconstruct the PPG signal for accurate HR evaluation. It should be noted that |*bpm_track_NF* − *bpm_prev*| > *th_NF_1* (a constant threshold) and |*bpm_track_EEMD* − *bpm_prev*| > *th_EEMD_1* after the **Ternary Decision 1**, |*bpm_track_NF* − *bpm_prev*| > *th_NF_2* (a constant threshold) and |*bpm_track_EEMD* − *bpm_prev*| > *th_EEMD_2* after the **Ternary Decision 2**. *th_NF* and *th_EEMD* are the empirical values obtained from the first 12 experiments.

**Figure 4:**
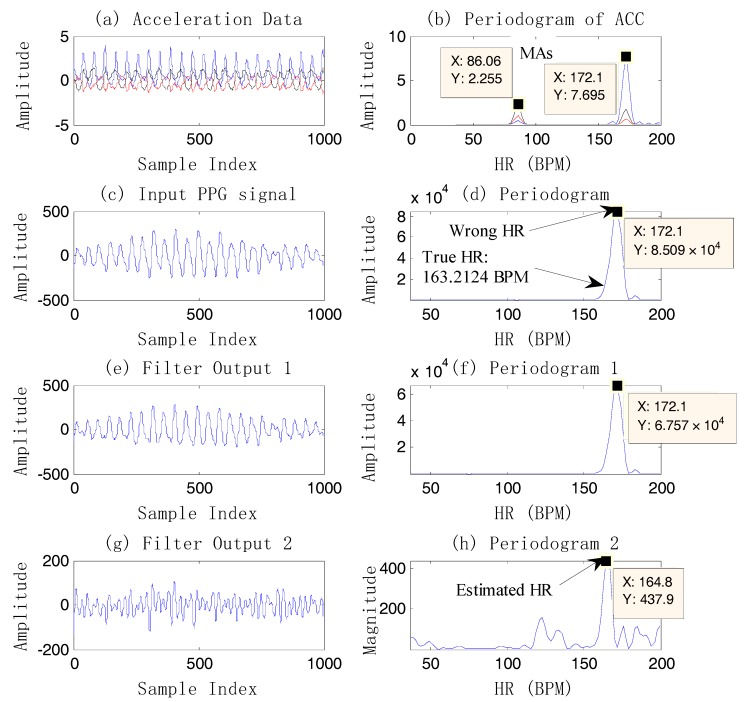
An example of HR estimation using the single notch filter on dataset 12. (**a**) Original acceleration data; (**b**) the periodogram of acceleration data; (**c**) the original PPG signal; (**d**) the periodogram of the PPG; (**e**) the reconstructed signal after the first single notch filter at 1.43 Hz (86.06 BPM); (**f**) the periodogram of the output signal 1 by filtering; (**g**) the final reconstructed signal after the second single notch filter at 2.87 Hz (172.1 BPM); (**h**) the periodogram of the output signal 2 by filtering.

It should be noted that the usage number of the repeated single notch filter is different in a different signal window. On the one hand, this number depends on all spectral peaks detected in the tri-axis acceleration signal spectrum. If there is a repeated value, it is recorded as one. On the other hand, if the number does not exceed three, then this number is the times the single notch filter is applied, and the frequencies of the notch are the detected peaks. If the number exceeds three, then a spectral peak value is found from the spectrum of each axis acceleration data; that is, the number of times the single notch filter is used is up to three. The purpose of limiting the number of application times is to retain the component corresponding to heart rate of the original signal as much as possible. Figure 4 shows an example of case 3.

From Figure 4, it is known that the final heart rate estimation after the NFEEMD algorithm is 164.8 BPM. The true HR value by ECG is 163.2 BPM. The absolute error is 1.6 BPM. In fact, we can find the only spectral peak, 172.1 BPM, in the periodogram of the original PPG signal. However, this value is quite different from the true HR. It is the effect of strong motion artifacts that results in a large error in HR estimation. It is worth noting that MAs are superimposed on the PPG signal closely in this example, so that the true HR information is covered by MAs. Although MAs conceal the true HR information, the tri-axis motion acceleration data accurately reflects the frequency components of motion artifacts. After removing the MAs by the single notch filter, the estimated HR value calculated from the final reconstructed signal is 164.8 BPM, which is very close to the true heart rate value. It is shown that using the periodogram directly may lead to a large error and lost HR tracking. This example also demonstrates that the repeated use of the single notch filter can accurately remove the MA components without affecting the nearby real HR components, and the HR-value resolution is high.

(2) Improved Ensemble Empirical Mode Decomposition

Empirical mode decomposition (EMD) is a kind of non-linear and non-stationary signal decomposition algorithm proposed by Professor Huang, which aims to decompose any complicated time-series signal into a number of “intrinsic mode functions” [18]. However, its drawbacks are obvious. End effect and mode mixing are the primary factors that can limit the development of EMD. On the basis of EMD, Wu et al. proposed a noise-assisted data analysis method, Ensemble Empirical Mode Decomposition (EEMD) [19]. In this algorithm, mirror continuation is used to remove end effect, and its key idea is that the mode-mixing phenomenon can be weakened by adding white Gaussian noise. Zhang et al. [17] and Khan et al. [20] have proven that EMD or the EEMD algorithm performance well in the analysis of biomedical signals.

In the EMD approach, the given original signal x(n) is decomposed into the IMF components and a residue. Each IMF component should satisfy two conditions: (1) the number of extrema and the number of zero-crossings must either be the same or differ at most by 1; and (2) the mean value of the upper envelope and lower envelope must be zero at any positions in the signal. EMD uses a “sifting process” to obtain the local zero-mean until an IMF and residue are found. The “sifting process” includes several steps as described follows: (1) identifying all extremes of x(n); (2) interpolating the upper envelope of maxima and the lower envelop of minima; (3) computing the mean of upper and lower envelope m(n); (4) extracting the detail c(n)=x(n)−m(n) and the residual; (5) iterating on the residual until c(n) satisfies the IMF conditions and then c(n) will be an IMF component; (6) repeating on the residual using sifting process (1)–(5) until the residual component is a monotonic function. The final decomposition result is described as follows:

(2) $$x(n)=\sum_{j=1}^{N}c_{j}(n)+r(n)$$

The updated EEMD algorithm adds an ensemble of *N_e_* signals into the given signal x(n) by adding white Gaussian noise $w_{p}(n)(p=1,2\ldots N_{e})$ which has the same variance. The equation can be described as follows:

(3) $$\tilde{x}(n)=x(n)+w_{p}(n),\;p=1,2\ldots N_{e}$$

Then, the EMD algorithm is applied to each of the new signals with noise, and the decomposition results can be described as follows:

(4) $$\tilde{x}(n)=\sum_{j=1}^{N}c_{pj}(n)+r_{p}(n),\;p=1,2\ldots N_{e}$$

Then, the optimum choice of IMFs is obtained by computing the average of an ensemble of *N_e_* signals of the EMD algorithm:

(5) $$c_{j}=\frac{1}{N_{e}}\sum_{p=1}^{N_{e}}c_{pj}(n),\;j=1,2\ldots N_{e}$$

The basic principle of EEMD based on noise-assisted analysis is that the time-frequency space is composed of different scale components divided by the filter group when the additional white noise is evenly distributed over the entire time-frequency space. Of course, EEMD cannot eliminate the effect of mode mixing completely due to the additional noise, especially when the parameter *N_e_* is small.

To make the reconstructed signal derived from EEMD cleaner, singular spectrum analysis (SSA) [11] is combined with EEMD to extract the usable reconstructed signal. SSA generates a trajectory matrix from the original signal by a sliding window of length L. The trajectory matrix is approximated using singular value decomposition (SVD). The last step is to reconstruct the series.

In this paper, SSA is applied to each IMF component after EEMD, and then the signal is reconstructed by only the first eigenvalue which corresponds to the most principal component over the whole signal. Finally, the only IMF whose frequency is closest to the previous heart rate frequency is chosen as the reconstructed signal.

Selecting which IMF belongs to the constructed signal is crucial. In this paper, the cycle of each IMF is first estimated. Then the IMF signal whose cycle is closest to the *bpm_prev* is chosen as the constructed signal.

**Figure 5:**
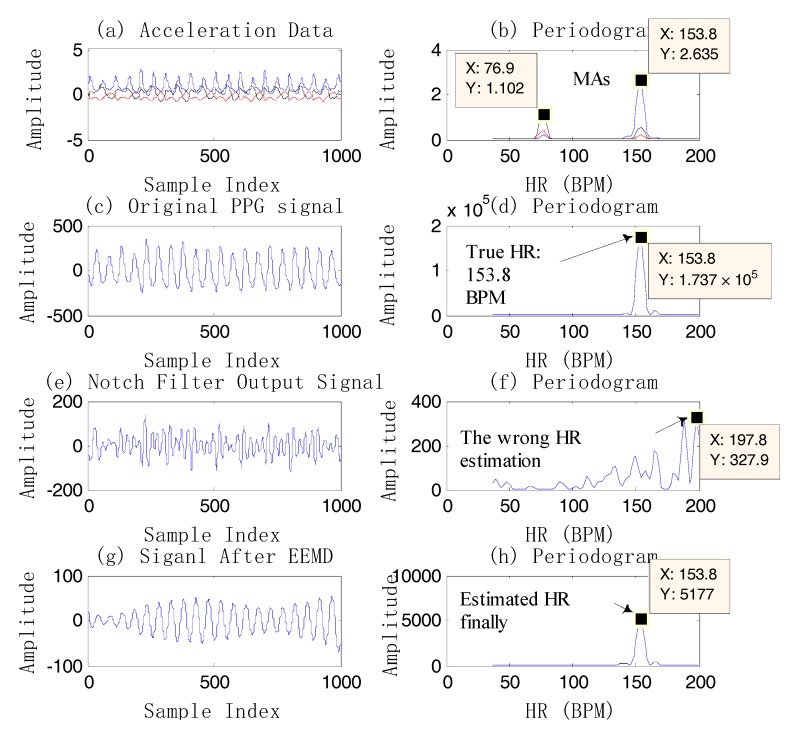
An example of HR estimation using EEMD on dataset 12. (**a**) Acceleration data; (**b**) the periodogram of acceleration data; (**c**) the original PPG signal; (**d**) the periodogram of PPG; (**e**) the output signal after two single notch filters at 1.28 Hz (76.9 BPM) and 2.56 Hz (153.8 BPM); (**f**) the periodogram of the output by filtering; (**g**) the reconstructed signal (IMF2) after EEMD (*bpm_prev* = 155.6 BPM); (**h**) the periodogram of the reconstructed signal.

In the example above, although the PPG signal is contaminated by MAs, the HR information is still obtained by the single notch filter. However, it is almost impossible to extract the HR information by the single notch filter when the PPG coincides with the motion artifacts (the spectral peak of the PPG is completely overlapped by the MA component.) (Figure 5e,f). So EEMD is another proposed method in this case.

**Figure 6:**
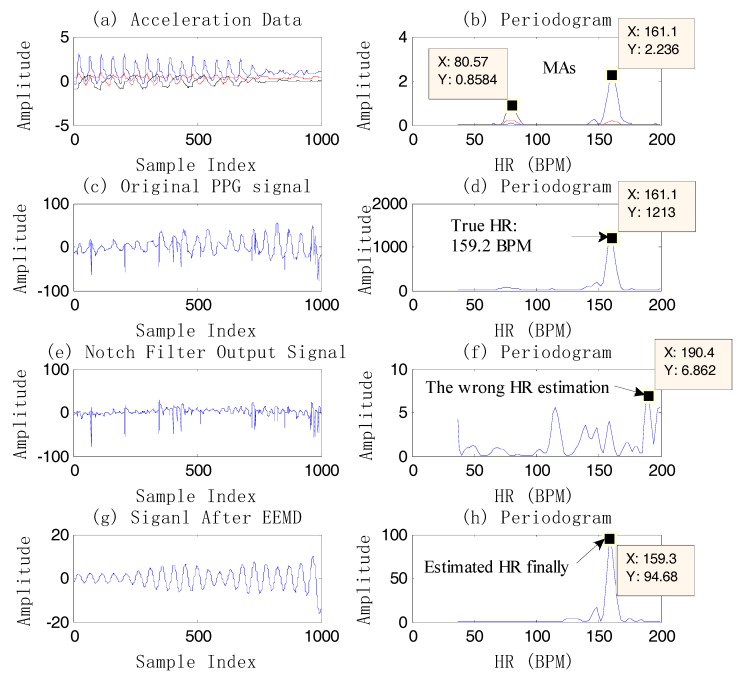
An example of HR estimation using EEMD on dataset 4. (**a**) Acceleration data; (**b**) the periodogram of acceleration data; (**c**) the original PPG signal; (**d**) the periodogram of the PPG; (**e**) the output signal after two single notch filters at 1.34 Hz (80.57 BPM) and 2.69 Hz (161.1 BPM); (**f**) the periodogram of the output signal by filtering; (**g**) the reconstructed signal (IMF4) after EEMD (*bpm_prev* = 159.3 BPM); (**h**) the periodogram of the reconstructed signal.

Figure 5 gives an example of HR estimation using EEMD instead of using the single notch filter. The final HR estimation value is 153.8 BPM, showing that EEMD is an effective method. It should be noted that the signal which has high signal quality in Figure 5g is the reconstructed one after the EEMD algorithm. From Figure 5, compared to the *bpm_prev*, the estimated HR by EEMD is closer to the true value, which shows the effectiveness of EEMD. On the other hand, there is a high-frequency resolution of estimation using EEMD, for example, Figure 6. The final estimated HR is 159.3 BPM, which is different from the spectral peak of the original PPG, 161.1 BPM.

**Case 4:** (the remaining one): The reason why this situation is carried out alone is that in this case the remaining frequency component is the most reliable. Therefore, if the absolute value of the heart-rate value corresponding to the remaining one and *bpm_prev* is less than *th_pass*, this value is considered acceptable. In other words, *bpm_track* = *bpm_prev*. According to experience, it is not necessary to use the above complex algorithm due to the low probability of it not being accepted in this case.

**Case 5:** (leaving more than one): In this circumstance, the heart rate (*bpm_track*) closest to the absolute value of the past is found. If |*bpm_track* − *bpm_prev*| < *th_pass*, then the value is accepted. Or *bpm_track* = *bpm_prev*.

**HR Calibration 2:** This is similar to the HR Calibration 1 mechanism. In fact, there is often a lack of tracking as the heart rate continues to rise. Therefore, HR calibration is employed mainly to solve this problem. On the one hand, the heart-rate value prediction of the current window is obtained through gray system prediction (GM (1, 1)) of the first 30 values when MAs exist. If the absolute value of the predicted value and the past value is greater than 12, then the next condition will be checked.

**Figure 7:**
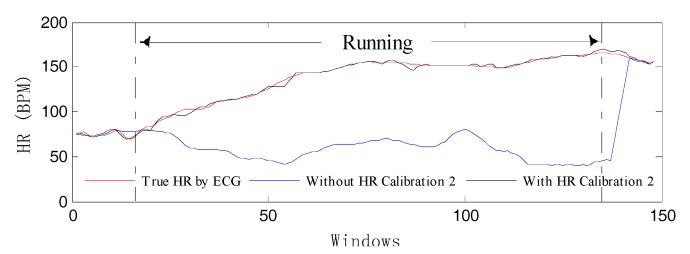
The algorithm performance with HR Calibration 2 and without HR Calibration 2 compared to the true HR by ECG.

On the other hand, three consecutive heart-rate values are dependent on the past. As long as these two conditions occur, the HR Calibration 1 algorithm will start when MAs exist to avoid losing track of spectral peaks. Then, a new *bpm_track* is calculated by the repeated single notch filter. If |*bpm_track* − *bpm_prev*| < 40, it is considered that *bpm_track* can be accepted. In order to better verify the effectiveness of the HR Calibration 2 model, we have done a set of comparative experiments to perform our algorithm in conditions using both HR Calibration 2 and not using it. Figure 7 illustrates the corresponding result, which shows that the HR-tracking trajectory (the blue curve) deviates from normal orbit when there is no calibration model. In contrast, the estimated HR (the black curve) can reflect the correct heart beat changes.

Additionally, the final output HR value of the current window is the average value of the previous windows by the cyclic moving average filter [16]. By virtue of the moving average filter with different windows, the final output result will be smooth.

## 3. Results

### 3.1. Database Introduction

To promote the development of HR estimation using PPG signals corrupted by intense motion artifacts, the IEEE Signal Processing Society has already organized an algorithm contest (IEEE Signal Processing Cup). Their datasets in the contest are also used in [10,11,12,13,14,15,17,21] and our NFEEMD algorithm also applies the same datasets. There are 23 datasets in total. It should be noted that the first 12 datasets are considered as the training data, and the last 11 datasets are considered as the test data in the following experiments.

Each of the first 12 training datasets (running on the treadmill) records two-channel PPG signals, three-axis acceleration signals, and one-channel ECG signals from subjects aged from 18 to 35. The last 11 test datasets (including wrist-intense exercise) record subjects aged from 19 to 58. For each subject, the PPG signals were recorded from the wrist by two pulse oximeters with green LEDs (wavelength: 515 nm). Their distance (from center to center) was 2 cm. The acceleration signal was also recorded from the wrist by a three-axis accelerometer. Both the pulse oximeter and the accelerometer were embedded in a wristband, which was comfortably worn. The ECG signal was recorded simultaneously from the chest using wet ECG sensors. All signals were sampled at 125 Hz and sent to a nearby computer via Bluetooth. Three types of activities were used in the experiments. T0 requires each subject to run on a treadmill with changing speeds. For datasets with names containing ‘TYPE01’, the running speeds changed as follows:

rest (30 s)→8 km/h (1 min)→15 km/h (1 min)→8 km/h (1 min)→15 km/h (1 min)→rest (30 s)

For datasets with names containing ‘TYPE02’, the running speeds changed as follows:

rest (30 s)→6 km/h (1 min)→12 km/h (1 min)→6 km/h (1 min)→12 km/h (1 min)→rest (30 s)

For the exercise type T1, the subject performed many actions including various forearm and upper arm exercises (e.g., shake hands, stretch, push, and so on, which are common in arm rehabilitation exercise), running, jumps, and push-ups. For the exercise type T2, the subject mainly performed intense forearm and upper arm movements (e.g., boxing). In each of this kind of datasets, heart rate is estimated in each time window of 8 s. Two successive time windows overlap by 6 s. Detailed experimental conditions follow in Table 1.

### 3.2. Performance Metrics

In this paper, the ground-truth HR from the simultaneous recorded ECG signal given by the database in each time window is chosen for comparison with the algorithm results. To evaluate the performance of the NFEEMD algorithm, two measurement indices which were also used in [10,11] are computed.

- AAE (Average Absolute Error)
  (6) $$AAE=\frac{1}{N}\sum_{i=1}^{N}\left|BPM_{est}(i)-BPM_{true}(i)\right|$$
  where $BPM_{est}\left(i\right)$ denotes the ground-truth HR value in the *i*-th time window; and $BPM_{true}\left(i\right)$ denotes the estimated HR using the NFEEMD algorithm.
- AEP (Absolute Error Percentage)
  (7) $$AEP=\frac{1}{N}\sum_{i=1}^{N}\frac{\left|BPM_{est}(i)-BPM_{true}(i)\right|}{BPM_{true}(i)}\times 100\%$$

In addition, the Bland–Altman plot is given to show the agreement between the ground-truth HR values and the estimated HR values. LOA (Limit of Agreement) is also calculated, which is defined by [μ−1.96σ,μ+1.96σ]. The last evaluation index is the Pearson correlation coefficient between the ground-truth HR and estimation.

### 3.3. Parameter-Setting and Experimental Results

In the NFEEMD algorithm, we set the number of FFT points of the periodogram to 4096, and apply a rectangle window. The sampling rate of PPG and ACC is 125 Hz. The ratio of the standard deviation of the added noise in EEMD is 0.05, and *N_e_* is 5. The window length of SSA is 150. *th_acc_peaks* is 5. *th_NF_*1 is 15 and *th_EEMD_*1 is 15 after **Ternary Decision 1**. *th_NF_*2 is 10 and *th_EEMD_*2 is 10 after ternary decision 2. *th_off* (a constant threshold) is 30 in case 3. *th_pass* is 8 in this paper. These thresholds are empirical values derived from the first 12 datasets.

In Table 2, the NFEEMD algorithm is compared with the other six recently-developed algorithms: TROIKA [11], JOSS [10], SpaMA [12], CNAFSD [13], SPECTRAP [14], and WFPV [15], via the average absolute error (AAE) and average absolute percentage (AEP).

## 4. Discussion

From Table 2, the NFEEMD algorithm performs better compared to the others. For the first 12 of 23 datasets, the average absolute error (AAE) is 1.12 + 0.51 (mean ± standard deviation) BPM, and AAE is 2.68 + 2.19 BPM for the remaining 11 datasets. For all 23 datasets, an average absolute error of 1.87 BPM and standard deviation of 1.79 BPM are recorded using the NFEEMD framework under intense physical activities.

It should be noted that the most obvious difference between the first 12 datasets and the last 11 datasets is the severity of motion. The activities of sample set T0 on the treadmill have a certain regularity, and the activities of sample set T1 and sample set T2 including arm movements are intense and random. In Table 2, the average absolute error of the last 11 datasets (2.68 BPM) by using NFEEMD is significantly larger than the first 12 datasets (1.12 BPM). This result is consistent with the severity of the state of motion, so the intenser the movements, the larger the HR-estimation error obtained. Although the errors are a bit larger for the last 11 datasets, HR estimates do not get derailed, which indicates that the direction of the HR estimates is correct. If the HR estimates get derailed for some reason in the process of estimation, our HR-calibration section could work (Figure 7, for example). As the results of comparisons in Table 2 show, the NFEEMD algorithm could obtain the most accurate results on HR estimates for the last 11 datasets, as well as the second most accurate results on HR estimates for the first 12 datasets. Compared with the SPECTRAP algorithm, the accuracy of the NFEEMD algorithm is slightly lower than the SPECTRAP for 22 datasets (except dataset 13). In a word, the results in Table 2 indicate that the NFEEMD algorithm can adapt to more intense circumstances like boxing in the last 11 recordings and our algorithm is more robust.

**Figure 8:**
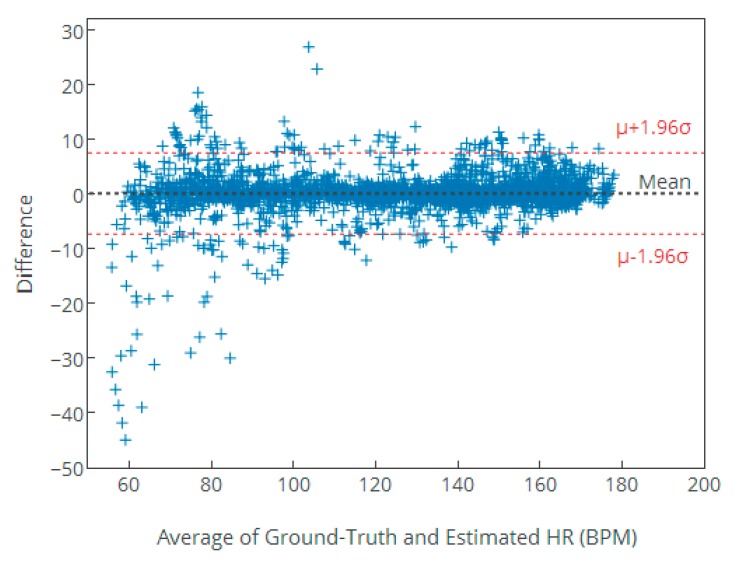
The Bland–Altman plot of the estimated HR values of the NFEEMD algorithm on the 23 datasets. The limit of agreement (LOA) is [−7.41, 7.45] BPM, where the absolute value of mean μ= 0.02 BPM, and standard deviation σ= 3.79 BPM.

The Bland–Altman plot is given in Figure 8 to test agreement between the ground-truth HR values and the estimation HR values, in order to show our algorithm’s better performance. From Figure 8, we can see that more than 95% of estimated HR values are incorporated in the limit of agreement (LOA) expressed by [μ−1.96σ, μ+1.96σ] where the absolute value of mean μ= 0.02 BPM and standard deviation σ= 3.79 BPM. The difference error is large when the real HR values are between 50 and 80, which indicates that several HR estimations using NFEEMD do not work well when the subject is in a stationary state except for intense arm exercise like boxing. The algorithm works well when HR is increasing continually, which indicates that the MA-removal method and the heart-rate tracking method are valid when the subject is in a sustained exercise state like running.

**Figure 9:**
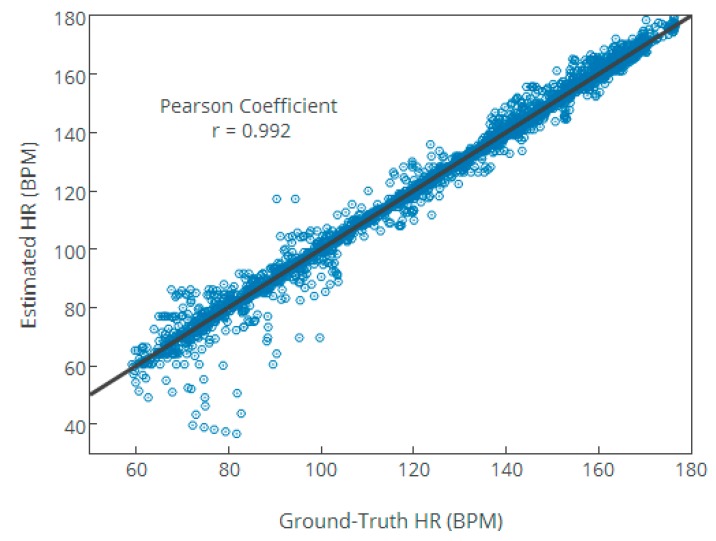
The scatter plot on the 23 datasets between the ground-truth and estimated HR values using the NFEEMD algorithm.

In addition, Figure 9 gives the scatter plot of 23 recordings between the ground-truth HR values and the estimated HR values, where the Pearson correlation coefficient is 0.992 and the fitted line is y=1.0101x−1.2454 (x is the ground-truth HR values and y is the estimated HR value). In Figure 9, the high Pearson coefficient and the small absolute value of mean indicate the NFEEMD algorithm’s better performance.

**Figure 10:**
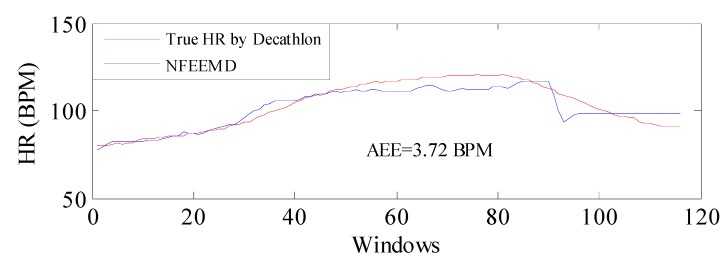
Performance of the NFEEMD algorithm for a new dataset collected by the wearable heart-rate monitoring equipment which is developed independently by our lab.

In order to verify the performance of the NFEEMD algorithm on HR tracking during intense physical exercise, we applied the proposed algorithm along with the same set of parameters to a new dataset where the subject performed intensive running and upper arm movements. The sampling frequency is 100 Hz. The results are given in Figure 10. It should be noted that the true HR values are obtained by the heart-rate monitor made by Decathlon and there would be errors in data obtained between this monitor and the ECG. From Figure 10, the average absolute error is 3.72 BPM, which indicates that the proposed algorithm performs well during intensive physical activities and the algorithm could be available for PPG derived from other platforms.

To examine in more detail the performance of the NFEEMD algorithm with a change in sampling frequency, we experimented in 25 Hz sampling frequency using the same algorithm for one channel PPG and three-channel ACC. The corresponding AAE results for all datasets are listed in Table 3, which demonstrates that the NFEEMD algorithm performs better in 125 Hz sampling frequency than in 25 Hz. In other words, more detailed information can be recorded at a high sampling frequency so that the HR-estimation accuracy can be improved.

Moreover, the signal sparsification technique through the M-FOCUSS in TROIKA and JOSS is applied on the HR estimation algorithm, which involves extensive computational complexity. For example, for the sampling frequency of 125 Hz, TROIKA takes about 3.5 h to estimate HR for the first 12 datasets on a computer equipped with Intel Core-i7 4790 at 3.6 GHz, 8-GB RAM, Windows 7 64 bit, and MATLAB 2013a. The algorithm proposed by Khan [20] takes 668 s on the same computer. The NFEEMD algorithm takes 229 s for calculation of the first 12 datasets and 476 s for all 23 datasets using the same computer configuration. In addition, our proposed algorithm takes 86 s for the first 12 datasets and 191 s for all 23 datasets when the sampling frequency is 25 Hz. JOSS takes 300 s for all the datasets at 25 Hz sampling frequency. It is obvious that our algorithm has the advantage of low computational complexity and short running time.

Of course, our algorithm also needs to be improved. On the one hand, Figure 8 shows that the difference error is large when the real HR values are between 50 and 80. The subjects tend to be in the rest state, or a critical state between rest and movement, when this circumstance occurs. In fact, HR estimation during running may be easier sometimes than in the rest or critical states, since in the latter process, there are large MA peaks and these peaks are actually scattered all over the spectrum. At this moment, there is no effective method to solve this problem. What is more, two-channel compared PPG signals can improve the algorithm performance and the correct HR peak can be determined with more confidence. On the other hand, high-frequency resolution and accuracy are obtained after using the repeated single notch filter advanced in this paper. Of course, an adaptive filter is also an option, although computation is increased. This research will be continued in future work.

## 5. Conclusions

This paper has proposed the NFEEMD HR estimation algorithm for intense physical exercises. One-channel PPG signal and tri-axis acceleration data are combined to generate a complex HR-estimation method. When MAs do not exist, only the spectrum of PPG is used to find the peak corresponding to HR information. However, the single notch filter and EEMD are applied to track the true HR value when MAs are strong. Finally, it is necessary to use the HR-calibration algorithm to avoid HR values heading into a runaway situation, so that the off-track errors can be decreased to a minimum. Tests on all of the 23 datasets of IEEE Signal Processing Society showed that the NFEEMD algorithm could comprehensively obtain higher accuracy than others, especially during intense physical activities. The measurement metrics illustrate that the NFEEMD algorithm is more robust and stable. This dynamic HR-estimation algorithm has many potential uses in wearable devices. People can monitor the real-time heart rate at home with wearable devices, or monitoring of long-term HR-tracking values can be undertaken for disease diagnosis.

## Figures and Tables

**Table 1:** 23 Datasets from IEEE Signal Processing.

ID	Dataset	Activity Type ^1^	Yrs	Weight/Height	Sex	Body Status
1	DATA_01_TYPE01	T0	18–35 y	None	M	Healthy
2–12	DATA_02_TYPE02–DATA_12_TYPE02	T0	18–35 y	None	M	Healthy
13	DATA_S04_T01	T1	20 y	64 kg/162 cm	M	Healthy
14	TEST_S01_T01	T1	29 y	70 kg/169 cm	M	Healthy
15	TEST_S02_T01	T1	21 y	77 kg/188 cm	M	Healthy
16	TEST_S02_T02	T2	21 y	77 kg/188 cm	M	Healthy
17	TEST_S03_T02	T2	19 y	54 kg/174 cm	M	Healthy
18	TEST_S04_T02	T2	20 y	64 kg/162 cm	M	Healthy
19	TEST_S05_T02	T2	20 y	57 kg/174 cm	M	Healthy
20	TEST_S06_T01	T1	19 y	70 kg/180 cm	M	Healthy
21	TEST_S06_T02	T2	19 y	70 kg/180 cm	M	Healthy
22	TEST_S07_T02	T2	21 y	73 kg/180 cm	M	Healthy
23	TEST_S08_T01	T1	58 y	70 kg/156 cm	F	Unhealthy ^2^

Activity type ^1^: T0 including resting/walking/running on a treadmill; T1 including arm rehabilitation exercise; T2 including intense arm movements (e.g., boxing); Unhealthy ^2^: Unhealthy means that the subject has abnormal heart rhythm and blood pressure.

**Table 2:** AAE and AEP results on 23 datasets of compared to other algorithms.

ID	Activity Type		TROIKA [11]	JOSS [10]	SpaMA [12]	CNAFSD [13]	SPECTRAP [14] (Offline)	WFPV [15]	[21]	NFEEMD (This Paper)
			AAE AEP%	AAE AEP%	AAE AEP%	AAE AEP%	AAE AEP%	AAE AEP%	AAE AEP%	AAE AEP%
1			2.29 2.18	1.33 1.19	1.23 1.14	1.66 1.42	1.18 1.04	1.25 1.15	1.72 1.50	1.43 1.19
2			2.19 2.37	1.75 1.66	1.59 1.30	1.56 1.44	2.42 2.33	1.41 1.30	1.33 1.30	1.15 1.03
3			2.00 1.50	1.47 1.27	0.57 0.45	0.65 0.53	0.86 0.66	0.71 0.59	0.90 0.75	0.75 0.59
4			2.15 2.00	1.48 1.41	0.44 0.31	1.48 1.51	1.38 1.31	0.97 0.88	1.28 1.20	1.24 1.12
5			2.01 1.22	0.69 0.51	0.47 0.31	0.77 0.60	0.92 0.74	0.75 0.57	0.93 0.69	0.91 0.68
6	T0		2.76 2.51	1.32 1.09	0.61 0.45	1.12 0.90	1.37 1.14	0.92 0.75	1.41 1.20	1.25 0.99
7			1.67 1.27	0.71 0.54	0.54 0.40	0.72 0.60	1.53 1.36	0.65 0.50	0.61 0.50	0.79 0.60
8			1.93 1.47	0.56 0.47	0.40 0.33	0.91 0.80	0.64 0.55	0.97 0.83	0.88 0.80	0.63 0.53
9			1.86 1.28	0.49 0.41	0.40 0.42	0.42 0.36	0.60 0.52	0.55 0.48	0.59 0.50	0.58 0.56
10			4.70 2.49	3.81 2.43	2.63 1.59	2.35 1.45	3.65 2.27	2.06 1.29	3.78 2.40	2.48 1.48
11			1.72 1.29	0.78 0.51	0.64 0.42	1.45 0.94	0.92 0.65	1.03 0.68	0.85 0.60	0.89 0.58
12			2.84 2.30	1.04 0.81	1.20 0.86	0.78 0.60	1.25 1.02	0.99 0.70	0.71 0.50	1.37 0.91
13			- -	- -	3.41 4.25	- -	- -	3.54 4.08	- -	3.20 3.59
14	T1		6.63 8.76	8.07 10.9	7.29 9.80	7.71 10.6	4.89 6.29	9.59 12.2	- -	8.64 11.3
15			1.94 2.56	1.61 2.01	2.73 2.21	1.62 2.02	1.58 1.98	2.57 3.16	- -	1.98 2.57
16			1.35 1.04	3.10 2.69	3.18 2.11	3.10 2.68	1.83 1.49	2.25 1.87	- -	1.47 1.14
17	T2		7.82 4.88	7.01 4.49	3.01 2.52	7.00 4.49	3.05 2.00	3.01 1.99	- -	1.95 1.10
18			2.46 2.00	2.99 2.52	4.46 3.23	2.99 2.52	1.62 1.36	2.73 2.29	- -	2.34 1.95
19			1.73 1.27	1.67 1.23	3.58 3.98	1.67 1.23	1.24 0.92	1.57 1.15	- -	1.47 1.08
20	T1		3.33 3.90	2.80 3.46	1.94 1.66	2.45 3.00	2.04 2.23	2.10 2.41	- -	3.22 3.66
21	T2		3.41 2.43	1.88 1.32	2.56 2.02	1.81 1.26	2.49 1.81	3.44 2.45	- -	3.54 2.49
22			2.69 2.12	0.92 0.74	3.12 3.28	0.92 0.74	1.16 0.92	1.61 1.26	- -	1.16 0.93
23	T1		0.51 0.59	0.49 0.57	1.72 1.97	0.49 0.57	0.66 0.79	0.75 0.88	- -	0.53 0.62
Mean ± SD	T0	AAE	2.34 + 0.83	1.28 + 0.90	0.89 + 0.60	1.16 + 0.55	1.50 + 0.86	1.02 + 0.41	1.25 + 0.87	1.12 + 0.51
	1–12	AEP%	1.82 + 0.53	1.01 + 0.61	0.67 + 0.44	0.93 + 0.42	1.12 + 0.61	0.81 + 0.29	1.00 + 0.56	0.86 + 0.31
	T1–T2	AAE	-	-	3.36 + 1.51	-	-	3.01 + 2.34	-	**2.68 + 2.19**
	13–23	AEP%	-	-	3.36 + 2.30	-	-	3.07 + 3.17	-	**2.76 + 3.01**
	Test	AAE	3.19 + 2.32	3.05 + 2.52	3.53 + 1.48	2.98 + 2.45	2.13 + 2.77	2.96 + 246	-	**2.63 + 2.30**
	14–23	AEP%	2.96 + 2.41	3.00 + 3.04	3.28 + 2.40	2.91 + 2.95	2.04 + 3.01	2.97 + 3.32	-	**2.68 + 3.16**
	1–12	AAE	2.78 + 1.67	2.09 + 1.99	2.01 + 1.70	1.98 + 1.90	1.79 + 1.87	1.90 + 1.91	-	1.81 + 1.73
	14–23	AEP%	2.34 + 1.73	1.92 + 2.27	1.85 + 2.09	1.83 + 2.20	1.52 + 1.22	1.79 + 2.44	-	1.68 + 2.27
	All	AAE	-	-	2.07 + 1.69	-	-	1.97 + 1.90	-	**1.87 + 1.71**
	1–23	AEP%	-	-	1.96 + 2.10	-	-	1.89 + 2.43	-	**1.77 ± 2.26**

**Table 3:** The AAE results of 23 datasets at 25 Hz sampling frequency.

Dataset	1	2	3	4	5	6	7	8
AAE(BPM)	1.79	1.52	0.82	1.45	1.09	1.35	1.20	0.51
Dataset	9	10	11	12	13	14	15	16
AAE(BPM)	0.74	1.95	1.00	1.77	3.39	10.99	3.10	1.94
Dataset	17	18	19	20	21	22	23	**Mean ± SD**
AAE(BPM)	3.62	2.69	1.94	2.80	4.65	2.44	0.50	**2.32 + 2.17**

## References

[1] Tamura T., Maeda Y., Sekine M., Yoshida M. (2014). Wearable photoplethysmographic sensors—Past and present. Electronics.

[2] Allen J. (2007). Photoplethysmography and its application in clinical physiological measurement. Phys. Meas..

[3] Chen Y., Li D., Li Y., Ma X., Wei J. (2017). Use moving average filter to reduce noises in wearable PPG during continuous monitoring. eHealth 360°.

[4] Lee B., Han J., Baek H.J., Shin J.H., Park K.S., Yi W.J. (2010). Improved elimination of motion artifacts from a photoplethysmographic signal using a kalman smoother with simultaneous accelerometry. Physiol. Meas..

[5] Raghuram M., Madhav K.V., Krishna E.H., Reddy K.A. Evaluation of Wavelets for Reduction of Motion Artifacts in Photoplethysmographic Signals. Proceedings of the 2010 10th International Conference on Information Sciences Signal Processing and their Applications (ISSPA).

[6] Reddy K.A., Kumar V.J. Motion Artifact Reduction in Photoplethysmographic Signals Using Singular Value Decomposition. Proceedings of the IMTC Instrumentation and Measurement Technology Conference.

[7] Sun X., Yang P., Li Y., Gao Z., Zhang Y.-T. Robust Heart Beat Detection from Photoplethysmography Interlaced with Motion Artifacts Based on Empirical Mode Decomposition. Proceedings of the 2012 IEEE-EMBS International Conference on Biomedical and Health Informatics (BHI).

[8] Kim B.S., Yoo S.K. (2006). Motion artifact reduction in photoplethysmography using independent component analysis. IEEE Trans. Biomed. Eng..

[9] Fukushima H., Kawanaka H., Bhuiyan M.S., Oguri K. Estimating Heart Rate Using Wrist-Type Photoplethysmography and Acceleration Sensor While Running. Proceedings of the 2012 Annual International Conference of the IEEE Engineering in Medicine and Biology Society (EMBC).

[10] Zhang Z. (2015). Photoplethysmography-based heart rate monitoring in physical activities via joint sparse spectrum reconstruction. IEEE Trans. Biomed. Eng..

[11] Zhang Z., Pi Z., Liu B. (2015). Troika: A general framework for heart rate monitoring using wrist-type photoplethysmographic signals during intensive physical exercise. IEEE Trans. Biomed. Eng..

[12] Salehizadeh S., Dao D., Bolkhovsky J., Cho C., Mendelson Y., Chon K.H. (2016). A novel time-varying spectral filtering algorithm for reconstruction of motion artifact corrupted heart rate signals during intense physical activities using a wearable photoplethysmogram sensor. Sensors.

[13] Ye Y., Cheng Y., He W., Hou M., Zhang Z. (2016). Combining nonlinear adaptive filtering and signal decomposition for motion artifact removal in wearable photoplethysmography. IEEE Sens. J..

[14] Sun B., Zhang Z. (2015). Photoplethysmography-based heart rate monitoring using asymmetric least squares spectrum subtraction and bayesian decision theory. IEEE Sens. J..

[15] Temko A. (2017). Accurate wearable heart rate monitoring during physical exercises using PPG. IEEE Trans. Biomed. Eng..

[16] Zhao D., Sun Y., Wan S., Wang F. (2017). Sfst: A robust framework for heart rate monitoring from photoplethysmography signals during physical activities. Biomed. Signal Process. Control.

[17] Zhang Y., Liu B., Zhang Z. (2015). Combining ensemble empirical mode decomposition with spectrum subtraction technique for heart rate monitoring using wrist-type photoplethysmography. Biomed. Signal Process. Control.

[18] Huang N.E., Shen Z., Long S.R., Wu M.C., Shih H.H., Zheng Q., Yen N.-C., Tung C.C., Liu H.H. (1998). In the Empirical Mode Decomposition and the Hilbert Spectrum for Nonlinear and Non-Stationary Time Series Analysis. Proceedings of the Royal Society of London A: Mathematical, Physical and Engineering Sciences.

[19] Wu Z., Huang N.E. (2009). Ensemble empirical mode decomposition: A noise-assisted data analysis method. Adv. Adapt. Data Anal..

[20] Khan E., Al Hossain F., Uddin S.Z., Alam S.K., Hasan M.K. (2016). A robust heart rate monitoring scheme using photoplethysmographic signals corrupted by intense motion artifacts. IEEE Trans. Biomed. Eng..

[21] Mashhadi M.B., Asadi E., Eskandari M., Kiani S., Marvasti F. (2015). Heart rate tracking using wrist-type photoplethysmographic (PPG) signals during physical exercise with simultaneous accelerometry. IEEE Signal Process. Lett..

